# 

**Published:** 1887-07-23

**Authors:** 


### THE HOSPITAL

An Institution, Family, and Parochial Journal of
Hospitals, Asylums, & all Agencies for the Care of the Sick, Criticism & News.

### CONTENTS.

**Wasted Holidays** - 273
**Words of Consolation.**—XLII. The Work of the Holy Ghost - 274
**A Good Prescription** - 274
**The Story of the Hospitals.**—Guy's Hospital - 275
**"More Light"** - 276
**The Doctor's Pulpit.**—Dangerous Drugs: Bromide of Potassium - 277
**The National Pension Fund** - 278
**Doctors in Fiction** - 279
**Notes and News.** — Hospital Appointments. — The After-Care Association. — The Hospitals Association Meetings. — A Thank-Offering. — Vacancies. — New Hospitals.—The Working Men and the Hospitals.—Annual Meetings and Reports.—The London School of Medicine for Women.—The Devonshire Hospital.—Scarlet Fever and School Fees.—Church Parade.—Grimsby Hospital and the Licensed Victuallers' Association.—The Working People's Hospital Fund.—Fête at the Dublin Royal Hospital for Incurables.—Fancy Bazaar in Aid of Northallerton Cottage Hospital 280
**Annotations.**—Dishonourable Consultants.—Untrained Bathers.—"Sir Oracle."—Lemon Juice - 283
**Till the Doctor Comes.**—XXII. Treatment of Emergencies - 284
**Disinfectants** - 284
**A Friendly Alias.** By W. J. E.—Chapter III - 285
**Incidents of Animal Life.**—Stories about Dogs - 287
**Children's Diarrhœa** - 287
**The Children's Corner.**—Puzzles, Answers, etc. - 288
**Amusements with Profit** - 288
**In- and Out-Patients' Hospital Diary** - 289

